# Anticancer activity of a thymidine quinoxaline conjugate is modulated by cytosolic thymidine pathways

**DOI:** 10.1186/s12885-015-1149-5

**Published:** 2015-03-21

**Authors:** Qiong Wei, Haijuan Liu, Honghao Zhou, Dejun Zhang, Zhiwei Zhang, Qibing Zhou

**Affiliations:** 1 Department of Nanomedicine & Biopharmaceuticals, National Engineering Research Center for Nanomedicine, Huazhong University of Science and Technology, Wuhan, Hubei China; 2 Hepatic Surgery Center, Tongji Hospital, Tongji Medical College, Huazhong University of Science and Technology, Wuhan, Hubei China; 3 Department of Medicinal Chemistry, Virginia Commonwealth University, Richmond, VA USA

**Keywords:** Thymidine conjugate, Thymidine phosphorylase, Thymidine kinase 1, Anticancer selectivity, Liver cancer

## Abstract

**Background:**

High levels of thymidine kinase 1 (TK1) and thymidine phosphorylase (TYMP) are key molecular targets by thymidine therapeutics in cancer treatment. The dual roles of TYMP as a tumor growth factor and a key activation enzyme of anticancer metabolites resulted in a mixed outcome in cancer patients. In this study, we investigated the roles of TK1 and TYMP on a thymidine quinoxaline conjugate to evaluate an alternative to circumvent the contradictive role of TYMP.

**Methods:**

TK1 and TYMP levels in multiple liver cell lines were assessed along with the cytotoxicity of the thymidine conjugate. Cellular accumulation of the thymidine conjugate was determined with organelle-specific dyes. The impacts of TK1 and TYMP were evaluated with siRNA/shRNA suppression and pseudoviral overexpression. Immunohistochemical analysis was performed on both normal and tumor tissues. In vivo study was carried out with a subcutaneous liver tumor model.

**Results:**

We found that the thymidine conjugate had varied activities in liver cancer cells with different levels of TK1 and TYMP. The conjugate mainly accumulated at endothelial reticulum and was consistent with cytosolic pathways. TK1 was responsible for the cytotoxicity yet high levels of TYMP counteracted such activities. Levels of TYMP and TK1 in the liver tumor tissues were significantly higher than those of normal liver tissues. Induced TK1 overexpression decreased the selectivity of dT-QX due to the concurring cytotoxicity in normal cells. In contrast, shRNA suppression of TYMP significantly enhanced the selective of the conjugate in vitro and reduced the tumor growth in vivo.

**Conclusions:**

TK1 was responsible for anticancer activity of dT-QX while levels of TYMP counteracted such an activity. The counteraction by TYMP could be overcome with RNA silencing to significantly enhance the dT-QX selectivity in cancer cells.

**Electronic supplementary material:**

The online version of this article (doi:10.1186/s12885-015-1149-5) contains supplementary material, which is available to authorized users.

## Background

Thymidine kinase 1 (TK1) and thymidine phosphorylase (TYMP) are key cytosolic thymidine salvage enzymes and targeted by anticancer thymidine therapeutics [1-5]. Two isoforms of TKs have been identified in cells, TK1 in cytosol and TK2 in mitochondria, which convert thymidine, 2’-deoxyuridine and 5-substiuted-2’-deoxyuridine or 2’-deoxycytidine (TK2) to the 5’-monophosphate form [2-4]. Low levels of TK1 are generally expressed in normal adult cells while high levels of TK1 are characteristic of cancer cells [6-9]. High levels of TYMP have been reported in the liver, lung and breast tumors and associate with poor prognostic outcome of cancer patients [6,10-13]. TYMP converts thymidine to thymine and 2-deoxyribose-1-phosphate reversibly as the catabolic pathway. Simultaneously, TYMP also acts as a platelet derived endothelial cell growth factor in tumor angiogenesis and metastasis [6,14-16]. The contradictive role of TYMP in cancer therapy refers to that high levels of TYMP are required for the activation of 5-fluorouracil prodrugs such as capecitabine to 5-fluoro-2’-deoxyuridine-5’-monophosphate (5-FdUMP) as the thymidylate synthase inhibitor via the reverse catabolic pathway, whereas high levels of TYMP at the same time act as the tumor growth factor [17-19]. Overexpression study has confirmed that although induced overexpression of TYMP gene resulted in enhanced responses to capecitabine, endothelial cell migration was simultaneously induced [20]. Due to the dual roles of TYMP, mixed and complex outcomes were reported in clinical trials of thymidine therapeutic [18,19,21,22]. For example, capecitabine or capecitabine combined with oxaliplatin resulted in only modest improvement in advanced hepatocellular carcinoma (HCC) patients [21,22]. 3’-Deoxy-3’-(18 F)-fluorothymidine in positron emission tomography is an effective contrast agent for the diagnosis of liver metastasis, fibrosarcoma and lung tumors [23,24]. However, the results did not correlate to the progressive levels of TK1 in tumor tissues, suggesting a complicated mechanism, possibly involving catabolism by TYMP [25,26].

**Figure 1:**
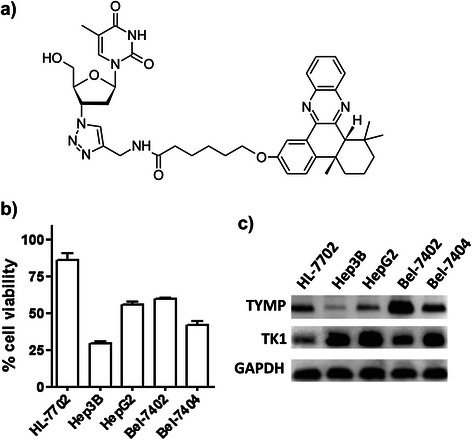
**Levels of dT-QX cytotoxicity and cellular TYMP and TK1 proteins among human liver cell lines. (a)** Chemical structure of thymidine analog dT-QX; **(b)** Cell viability MTT assay on human liver cell lines including HL-7702, Hep3B, HepG2, Bel-7402 and Bel-7404 after treatment of dT-QX at 50 μM for 24 h (Each data point in the graphs was the mean of triplicates with SEM); **(c)** Western blot analysis of TYMP and TK1 protein expression in HL-7702, Hep3B, HepG2, Bel-7402 and Bel-7404 liver cells.

One strategy to circumvent the dual roles of TYMP in anticancer thymidine therapeutic is to use cytotoxic thymidine analogs other than capecitabine that do not require metabolic activation by TYMP. For instance, 5-fluoro-2’-deoxyuridine (FdUrd) can be directly converted to active 5-FdUMP by the high levels of TK in cancer cells. Unfortunately, FdUrd shows far less anticancer potency with high toxicity due to the rapid catabolism by TYMP as compared with 5-fluorouracil and capecitabine [27,28]. Thus, a different selective cytotoxic thymidine analog would be needed. Recently, we reported a thymidine quinoxaline conjugate (dT-QX) with a broad spectrum of anticancer activity and low cytotoxicity on the normal liver cell line [29]. Although the selectivity of dT-QX was attributed to its unique thymidine linked chemical structure (Figure 1a), the molecular pathways responsible for the selectivity are unclear. Thus, dT-QX serves a chemical entity to investigate how TK1 and TYMP impact the activity in cancer cells. This may potentially reveal an alternative strategy for thymidine anticancer therapeutic to overcome the dual roles of TYMP. In this study, we reported the involvement of TK1 and TYMP in the biological activity of thymidine analog dT-QX in different liver cancer cell lines, methods to enhance the anticancer selectivity and in vivo study with a mouse tumor model.

## Methods

### Cells

Liver cancer cell lines Hep3B and HepG2 were obtained from American Type Culture Collection, USA. Human liver cells HL-7702 and liver cancer cells Bel-7402 and Bel-7404 were from Shanghai Institute of Life Science Cell Culture Center, China. Cells were maintained in high glucose DMEM medium (Invitrogen, USA) supplemented with 10% heat-inactivated fetal bovine serum, 25 mM HEPES, 2 mM L-glutamine, 0.1 mM nonessential amino acids, 1.0 mM sodium pyruvate, 50 U/mL penicillin, and 50 μg/mL streptomycin at 37°C and 5% CO_2_.

### Cell MTT viability assay

Cells were plated overnight at 5,000 per well on a 96-well plate and then treated with 50 μM dT-QX for 24 h in the growth media containing 10% serum and 0.1% DMSO. MTT assay was carried out as reported [29], and cell viability was plotted using GraphPad Prism software (GraphPad Software, USA). Thymidine analog dT-QX was synthesized as previously reported [29]. Stock solutions of dT-QX (50 mM) were prepared in DMSO and then diluted in water as a 10× treatment solution containing 0.1% tween-80 and 1% DMSO.

### Fluorescence study of dT-QX accumulation in cells

Cells were plated overnight at a density of 20,000 cells per well on a 48-well plate. For staining with organelle-specific fluorescent trackers, cells were treated with the DMSO control (0.1%) or dT-QX (50 μM) in the full growth media for 5 h. The treatment media were replaced with an endoplasmic reticulum (ER) Tracker Red staining solution (1 μM in PBS, Invitrogen, USA) or a mitochondrial MitoTracker Orange CMTMRos staining solution (100 nM in PBS, Invitrogen, USA) at 37°C for 30 min and then PBS. Live cell images were captured in PBS with Olympus IX71 inverted microscope (Tokyo, Japan) equipped with a digital camera under appropriate fluorescence filter sets. For ER-specific GFP expression, cells after plating overnight were transfected with BacMam ER Cell Light GFP reagent (6 μL, Invitrogen, USA). After 24 h, treatment of dT-QX or DMSO for 5 h were carried out similarly as described above. Live cell images were then captured in PBS with Olympus fluorescence microscope similarly.

### Western blot analysis

Cells were plated overnight at a density of 1 × 10^6^ cells per well on a 6-well plate. Cells were washed with PBS and lysed with 150 μL RIPA buffer containing protease and phosphatase inhibitor cocktail. The supernatants were collected by centrifuge at 14,000 g × 10 min at 4°C and stored at −80°C. The total protein content in lysates was determined by enhanced BCA protein assay kit (Beyotime Institute of Biotechnology, China). Electrophoresis was carried out on NuPAGE Novex Bis-Tris 4-14% gel (Invitrogen, USA) under the reduced condition with 5 μg of proteins per lane. The membrane was incubated with rabbit anti-TK1 monoclonal (ab76495, Abcam, USA) or anti-TYMP polyclonal antibody (ab69120, Abcam, USA) and mouse GAPDH antibody (Invitrogen, USA). Targeted proteins were visualized with Qdot 625 conjugate kit (Invitrogen, USA). Gel images were captured with ZF-258 Gel Imaging System (Shanghai Jiapeng Scientific Co. Ltd, China) under illuminating light of 350 nm wavelength.

### siRNA suppression study

Suppression of TK1 or TYMP proteins in cells was performed using Amaxa Nucleofector Kit V with program T-028 (Lonza, Germany) and Silencer-select Validated siRNAs for human TK1 (100 nM, 1:1 mixture of s14158 and s14159, Ambion, USA), TYMP (400 nM, s4433) or control (100 nM) according to manufacturer’s protocol. After electroporation, cells were plated for 40 h and then divided into two portions for western blot and MTT viability studies. Western blot analysis of TK1 and TYMP in cells were carried out at 48 h post transfection as described above. For MTT study, cells were treated with 0, 10, 20 or 50 μM of dT-QX in the growth media at 48 h post transfection. Cell viability MTT assay was carried out after treatment for 24 h and analyzed with GraphPad Prism software (GraphPad Software, USA).

### Transduction with TK1 lentiviral particles

A lentiviral open reading frame plasmid Lv-TK1 (EX-C0529-Lv105) containing human TK1 mRNA complete sequence [PubMed cDNA clone MGC number: 3644] and a control plasmid (EX-NEG-Lv105) were obtained from GeneCopoeia, USA. The sequences of cloned plasmids were confirmed by DNA sequencing using 5’-GCGGT AGGCG TGTAC GGT and 5’-ATTGT GGATG AATAC TGCC as the forward and reverse primers, respectively. Pseudo lentiviral particles for TK1 overexpression and the control were produced with Lv-TK1 or the control plasmid and Lenti-Pac HIV expression packaging kit (GeneCopoeia, USA) on 293T cells according to manufacturer’s protocol. Pseudovirus titer was estimated on Hep3B cells under the selection of puromycin (0.5 μg/mL) as 1.6 × 10^7^ and 1.2 × 10^7^ transducing units/mL for TK1 and the control, respectively. Transduction of HL-7702 and Bel-7402 cells was carried out at a cell density of 50,000 in a 24-well plate with 80 μL pseudovirus stock solution plus poloxamer F108 (100 μg/mL, 10 μL) and polybrene 100 (100 μg/mL, 10 μL). After 24 h, the transduction media were replaced with the normal DMEM growth media, and cells were grown in a 6-well plate for 7 days. Western blot analysis and cell viability MTT assay with compound treatment were then carried out similarly as described in siRNA study.

### shRNA suppression of TYMP

Suppression of TYMP in cells was performed with Amaxa Nucleofector Kit V with program T-028 (Lonza, Germany) and SureSilencing shRNA TYMP plasmid (25 μg, KH02651P, clone No 4, Qiagen, USA) or the ontrol plasmid (25 μg, NEG4-P). The inserted sequence in shRNA TYMP plasmid was confirmed by DNA sequencing. After electroporation, cells were plated for 60 h and then divided into two portions for western blot and MTT viability studies. Western blot analysis of TK1 and TYMP levels in cells and cell viability MTT assay were carried out at 72 h post electroporation similarly as described above.

### Immunohistochemical analysis

The use of human pathological tissue slides was approved by the Medical Ethnical Committee of Huazhong University of Science and Technology. Immunohistochemical (IHC) analysis was carried out according to manufacturer’s recommendation. Briefly, after deparafinization, antigen retrieval and protein block, tissue section slides were incubated with rabbit TK1 (ab59271, Abcam, USA) or TYMP polyclonal antibody (ab69120, Abcam, USA) in Tris saline buffer with 1% BSA (1:200 dilution). Staining was achieved using rabbit specific HRP/DAB detection IHC kit (ab64261, Abcam, USA). Staining of nuclei was carried out with a hematoxylin solution. Slides were mounted, and images were captured with Olympus IX71 inverted microscope (Tokyo, Japan).

### Mouse tumor model study

Animal protocol was approved by the Animal Care and Use Committee of College of Life Science and Technology at Huazhong University of Science and Technology. SFP male nude BALB/c mice (approximately 24 g) were obtained from Hunan Slake Jingda Experimental Animal Co. Ltd., China. Human liver cancer Bel-7402 cells (1x10^7^ cells per mouse) were injected subcutaneously at the lower back of nude BALB/c mice [30]. Once the tumor reached to an average size of 9 × 9 mm, mice were randomly divided into groups for the following studies. In vivo transfection control and TYMP shRNA plasmid complex (200 μL) were prepared in a sterile 5% glucose solution with TurboFect agent (ThermoFisher, USA) and injected intratumorally at a dose of 10 μg DNA, 50 μL per mouse. In vivo suppression of TYMP in tumor tissue via intratumoral injection was first validated by western blot analysis. After 72 h, mice were euthanized and tumor tissues were collected and homogenized at 4°C in 750 μL RIPA lysis buffer containing protease and phosphatase inhibitor cocktail. The supernatants were collected by centrifuge at 14,000 g × 10 min at 4°C, and western blot analysis was carried out as described above. Treatment groups included: a) iv injection of PBS for 4 day (3 mice); b) iv injection of dT-QX (0.75 mg/kg body weight) for 4 days (3 mice); c) intratumoral injection of TYMP shRNA followed by iv injection of PBS after 2 days for 4 days (4 mice); and d) intratumoral injection of TYMP shRNA followed by iv injection of dT-QX (0.75 mg/kg body weight) after 2 days for 4 days (4 mice). The treatment was repeated one more time on day 8. During the treatment, the growth of tumors and body weight were monitored daily. Statistical analysis of the treatments was performed with GraphPad Prism software using two way ANOVA with Bonferroni posttests. No significant abnormal behavior or weight loss was observed throughout the treatment. Images of tumors were obtained on day 18 at the end of treatment study.

## Results

### dT-QX exhibits varied cytotoxicity on liver cancer cells that have different levels of TK1 and TYMP

In addition to the reported selective activity of dT-QX [29], significant variation in the cytotoxicity of thymidine analog dT-QX was found among five different liver cell lines, with 70% for Hep3B cells, 60% for Bel-7404 cells, and down to 45% and 40% for HepG2 and Bel-7402, respectively after 24 h incubation. In contrast, only 14% cytotoxicity was observed in HL-7702 cell line (Figure 1b). Because dT-QX is an analog of thymidine, levels of key thymidine salvage and metabolic enzymes such as TYMP and TK1 in these cells were investigated to see whether there was any correlation to the levels of the cytotoxicity. Western blot analysis revealed that there was a significant contrast in the levels of TYMP and TK1 among these cell lines (Figure 1c). For catabolic TYMP, only a basal level was found in Hep3B cells while low levels of expression were observed in HL-7702, HepG2 and Bel-7404 cells. In contrast, Bel-7402 cells had significantly high levels of TYMP. Simultaneously, TK1 was highly expressed in Hep3B, HepG2 and Bel-7404 cells, intermediately in Bel-7402 and minimal in HL-7702 cells. These results implied that the cell toxicity of dT-QX might correlate with TK1 levels in cells, i.e., high in Hep3B and Bel-7404 cells, intermediate in Bel-7402 and low in HL-7702 cells except that HepG2 cells did not fit well with this hypothesis. The role of TYMP on the biological activity of dT-QX was not clear based on these data. The possible correlation of high levels of TK1 with the cytotoxicity of dT-QX suggested that dT-QX might be significantly converted to the 5’-phosphate form in Hep3B cells by the salvage pathway as a thymidine analog. Thus, HPLC analysis of the Hep3B cells lysate after treatment of 50 μM dT-QX was carried out under various conditions with HPLC separation conditions for nucleosides and nucleotides [31]. Unfortunately, only intact dT-QX was observed in HPLC analysis based on the unique UV absorbance signals of dT-QX at 365 nm coupled with mass analysis. Therefore, the roles of thymidine pathways on the dT-QX cytotoxicity needed to be determined and were investigated by the following alternative methods.

**Figure 2:**
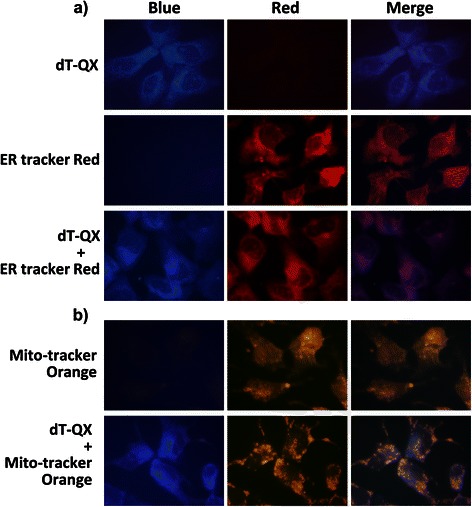
**Fluorescence images of intracellular accumulation of dT-QX in Hep3B cells.** Cells were treated with 50 μM dT-QX or DMSO for 5 h and then stained with organelle-specific ER Tractor Red **(a)** or Mito-tracker Orange fluorescent dye **(b)**. Images were representative from three independent studies.

The cellular accumulation of dT-QX was first assessed by the fluorescent property of dT-QX. dT-QX has a maximum excitation and emission at 398 and 483 nm, respectively [see Additional file 1: Figure S1], similar to the fluorescent dye Hoechst 34580 [32]. It was implied that dT-QX accumulated mostly at ER in Hep3B cells because the blue fluorescence of dT-QX matched with most of ER-specific red fluorescence in cells (Figure 2a). Cellular accumulation of dT-QX was also compared with mitochondria-specific fluorescent tracker because mitochondrial TK2 could also phosphorylate thymidine analogs as an additional salvage pathway besides cytosolic TK1 [33,34]. The resulting images suggested that dT-QX accumulated at sites other than the mitochondria (Figure 2b), although there were some overlaps of dT-QX in the mitochondria. Further studies with HepG2 and HL-7702 cells also indicated a similar ER accumulation of dT-QX by ER-specific tracker [see Additional file 1: Figure S1]. Complementary ER-specific GFP expression using transfection method also consistently suggested ER as the major accumulation site of dT-QX [see Additional file 1: Figure S1]. In addition, the ER location of dT-QX in cells did not change significantly with extended incubation time over 10 h. These results suggested that the cytotoxicity of dT-QX were modulated via cytosolic processes.

Significant inhibition of DNA synthesis in cells has been previously reported upon the treatment of Hep3B and HepG2 cells with dT-QX for 5 h, but not in HL-7702 cells with low TK1 and TYMP expression [29]. On the other hand, the cellular accumulation of dT-QX was only observed in the cytosol not in the nuclei of cells (Figure 2). In addition, no 5’-phoaphate metabolite of dT-QX was found by HPLC analysis with Hep3B cell lysates in this study. These results presented a dilemma on the mechanism of dT-QX because phosphorylated dT-QX was expected to be formed and observed in the nuclei of cancer cells. The key question would be whether TK1 and TYMP were involved in the cytotoxic action of dT-QX in cancer cells. Thus, we focused on the following investigations on the cell-based study to assess the impact of TK1 and TYMP on the activity of dT-QX, rather than the purified recombinant TK1 and TYMP protein study.

### Cellular TK1 and TYMP levels regulate the selective cytotoxicity of dT-QX

**Figure 3:**
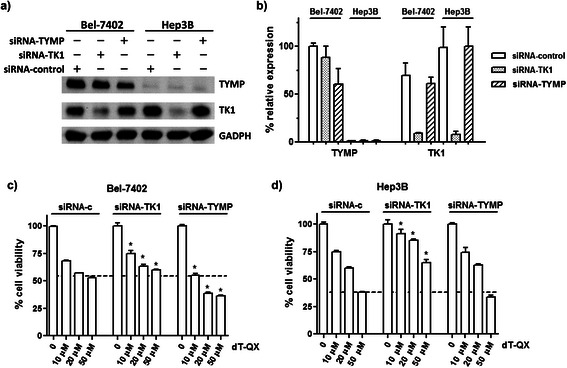
**Silence of TK1 or TYMP expression impacted dT-QX cytotoxicity. (a)** Western blot analysis of TYMP and TK1 in Bel-7402 and Hep3B cells at 48 h post siRNA suppression; **(b)** Relative percent protein expression of TYMP and TK1 in western blot analysis after normalization with that of GADPH; **(c)** and **(d)** Cell viability MTT results after 24 h treatment with dT-QX at 48 h post siRNA suppression in Bel-7402 and Hep3B cells. Each data point in the graphs was the mean of triplicates with SEM. All experiments were independently repeated at least two times (*P < 0.05 as compared to those under the same dT-QX concentration in controls).

Investigation of the involvement of TYMP and TK1 in the cytotoxicity of dT-QX was verified with transient siRNA silencing of either protein followed by MTT viability assay after dT-QX treatment. Hep3B and Bel-7402 cells were investigated as representatives because TK1 protein was predominantly expressed in Hep3B cells with basal levels of TYMP while Bel-7402 has the highest level of TYMP (Figure 1c). Upon transient silencing TK1 protein in Hep3B cells, the cell viability was markedly increased at all concentrations compared with those of siRNA-control, e.g., from 38% to 65% at 50 μM dT-QX (Figure 3d). Increase of the cell viability was similarly observed in Bel-7402 cells, although at a less extent (Figure 3c). In contrast, a reduced TYMP level in Bel-7702 cells led to a pronounced decrease of cell viability by 15% (Figure 3c). These results implied that TK1 was involved in the cytotoxicity of dT-QX and that high levels of TYMP counteracted the biological activity of dT-QX in cells. This observance was also consistent with the low cytotoxicity of dT-QX observed in HL-7702 cells where neither TK1 nor TYMP was significantly expressed (Figure 1c). Similarly, for HepG2 cells, siRNA silencing study showed that transient suppression of TK1 led to a significant decrease of dT-QX cytotoxicity at all concentrations whereas silencing TYMP produced a 7% increase of dT-QX activity at 50 μM [see Additional file 2: Figure S2].

**Figure 4:**
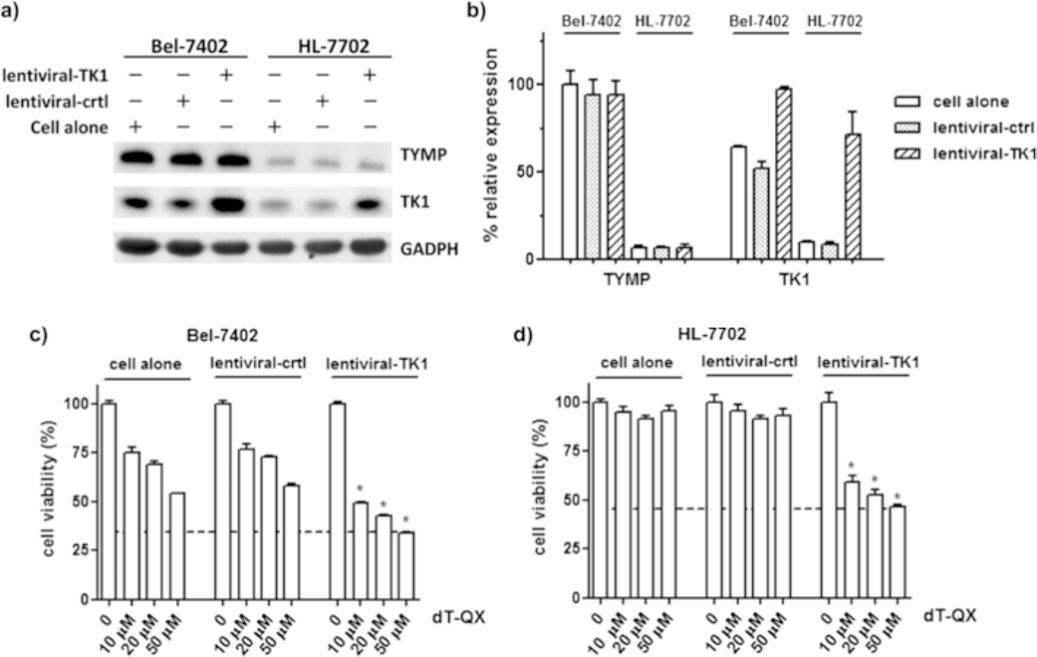
**Viral overexpression of TK1 enhanced the cytotoxicity of dT-QX. (a)** Western blot analysis of TYMP and TK1 level post transduction of Bel-7402 and HL-7702 cells with either control or TK1 pseudo lentiviral particles; **(b)** Relative percent protein expression of TYMP and TK1 in western blot analysis after normalization with that of GADPH; **(c)** and **(d)** Cell viability MTT results after 24 h treatment with dT-QX post viral transduction in Bel-7402 and HL-7702 cells. Each data point in the graphs was the mean of triplicates with SEM. All experiments were independently repeated at least two times (*P < 0.05 as compared to those under the same dT-QX concentration in cells alone).

To further confirm TK1 were mainly responsible for the dT-QX cytotoxicity in cells, lentiviral overexpression of TK1 was carried out on Bel-7402 cells and HL-7702 cell line as a comparison (Figure 4). The pseudo lentiviral viral particles can deliver and integrate a human TK1 gene into the genome of targeted cells without virus replication. Western blot analysis confirmed that the overexpression of TK1 was achieved in Bel-7402 cells by 1.5 folds and 9 folds in HL-7702 cells (Figure 4a-b). Consistently with TK1 activation, the cytotoxicity of dT-QX increased by 20% at all concentrations from 10 to 50 μM versus those of Bel-7402 cells alone (Figure 4c). More importantly, a phenomenal cytotoxicity of dT-QX was observed in HL-7702 cells with lentiviral overexpression of TK1 versus those of cells alone (Figure 4d). Therefore, the results from our siRNA suppression and viral overexpression studies indicated that high levels of cytosolic TK1 were responsible for the cytotoxicity of dT-QX in liver cancer cells while high levels of TYMP counteracted the biological activity.

### High levels of TYMP are an important clinical subtype and can effectively be counteracted by shRNA silencing

**Figure 5:**
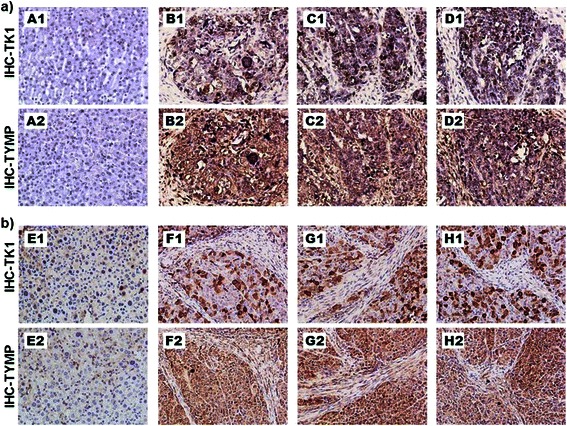
**High levels of TYMP and TK1 are clinically significant in liver tumor tissues. (a)** IHC analysis of TK1 (top) and TYMP (below) on human normal liver tissue sample (A) and human liver cancer tumor tissue sample (B, C and D) co-stained with hematoxylin; **(b)** IHC analysis of TK1 (top panels) and TYMP (bottom panels) on mouse normal liver tissue (E) and mouse Bel-7402 tumor tissue (F, G and H) co-stained with hematoxylin.

Clinical relevance of high levels of TK1 and TYMP was then assessed on human HCC tumor and normal liver tissues with immunohistochemical (IHC) analysis. Normal human liver tissue showed only low basal levels of TYMP and TK1 protein expression as compared to those of tumor samples (panel A versus B, C and D, Figure 5a). This result validated that HL-7702 cells with low levels of TYMP and TYMP was a derived normal liver cell line for this study (Figure 1c). In contrast, TYMP and TK1 positive staining were overwhelmingly observed in the tumor tissues, suggesting that Bel-7402 cell line indeed represented such a subtype of liver tumors. These results indicated that high expression of TYMP and TK1 in liver tumors was an important subtype of liver cancers that was needed to be addressed specifically for anti-cancer thymidine analog dT-QX. Moreover, induced high level of TYMP has been found in tumor tissues due to inflammatory infiltration or after radiotherapeutic treatment and chemotherapy such as paclitaxel, doxorubicin and oxaliplatin [18,35,36]. Furthermore, tumor tissues from the established Bel-7402 mouse model had consistently high expression of TYMP and TK1 by IHC analysis, whereas the mouse normal liver tissue showed only basal level of either protein (panels F-H versus panels E, Figure 5b). Thus, the Bel-7402 mouse tumor model was validated and used for the following in vivo dT-QX treatment study.

**Figure 6:**
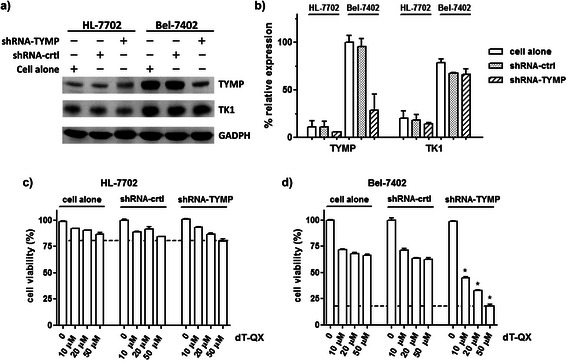
**ShRNA suppression of TYMP was effective to enhance the selective cytotoxicity of dT-QX. (a)** Western blot analysis of TYMP and TK1 level at 72 h post transfection of HL-7702 and Bel-7402 cells with either the control or TYMP shRNA plasmid; **(b)** Relative percent protein expression of TYMP and TK1 in western blot analysis after normalization with that of GADPH; **(c)** and **(d)** Cell viability MTT results after 24 h treatment with dT-QX at 72 h post shRNA suppression in Bel-7402 and Hep3B cells. Each data point in the graphs was the mean of triplicates with SEM. All experiments were independently repeated at least two times (*P < 0.05 as compared to those under the same dT-QX concentration in cells alone).

To enhance the selective cytotoxicity of dT-QX, viral overexpression of TK1 in cells clearly was not an effective strategy on cancer cells due to a concurring high cytotoxicity in normal liver HL-7702 cells (Figure 4). Alternative way was to significantly knock down the TYMP level as indicated with siRNA suppression (Figure 3). However, siRNA suppression was not effective to significantly lower TYMP level in Bel-7402 cells even at a high concentration of 400 nM (Figure 3a-b). Recently, shRNA silencing has been shown to be an effective method for both *in vitro* cellular and *in vivo* animal studies [37]. Thus, transfection of shRNA TYMP plasmid on Bel-7402 was carried out. Western blot analysis confirmed that approximately 70% suppression of TYMP was achieved in Bel-7402 cells while the level of TK1 was not impacted (Figure 6a-b). Subsequent cell viability study revealed a significantly elevated cytotoxicity of dT-QX versus those of cells alone. In contrast, no impact on TYMP or TK1 was found in HL-7702 cells under the same condition. More importantly, no significant cytotoxicity was observed in HL-7702 cells (Figure 6c). All these results indicated that suppression of TYMP by shRNA is an effective approach to enhance the selective cytotoxicity of dT-QX on cancer cells with high levels of TYMP and TK1.

### Combination of TYMP suppression plus dT-QX treatment is effective in the liver tumor model in vivo

**Figure 7:**
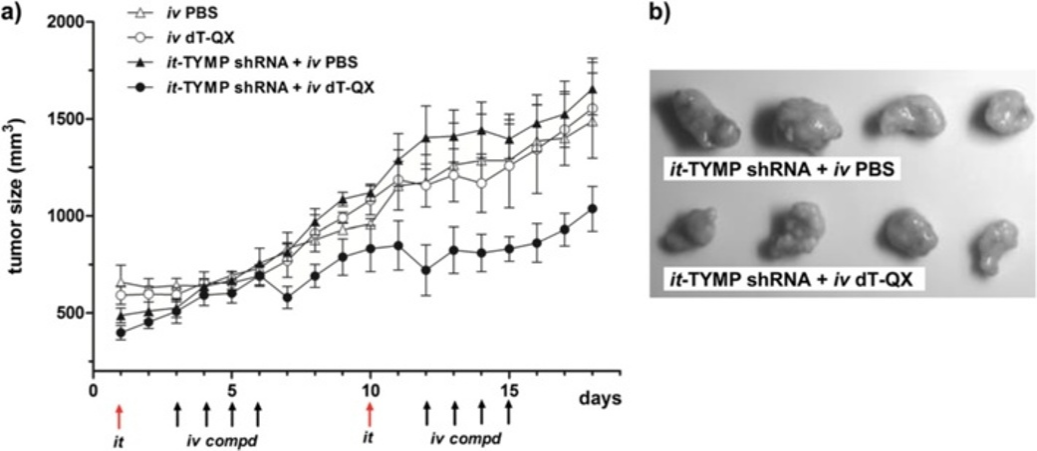
**In vivo study of TYMP shRNA plus dT-QX treatment in the subcutaneous Bel-7402 mouse tumor model. (a)** Growth profile of the tumor size over 2 repeated treatment with or without intratumoral injection of TYMP shRNA followed by intravenous injection of dT-QX or PBS; Statistical analysis of the treatments was performed with GraphPad Prism software using two way ANOVA with Bonferroni posttests, indicating that there was significant difference over time between the group of it-TYMP shRNA + iv dT-QX and other three groups (P < 0.05). **(b)** Images of the tumors after 2 repeated treatment with intratumoral shRNA injection.

In vivo validation of the combined treatment of TYMP shRNA suppression plus dT-QX was carried out in a subcutaneous tumor model of human liver cancer Bel-7402 cells. Western blot analysis indicated that intratumoral injection of TYMP shRNA complex in vivo significantly reduced the TYMP level in tumor tissue than those of control at 72 h post injection [see Additional file 3: Figure S3], confirming the effectiveness of intratumoral delivery of shRNA. The combined treatment was then carried out in the tumor model with the intratumoral delivery of TYMP shRNA complex first and then intravenous injection of dT-QX or PBS (Figure 7). Clearly, TYMP shRNA plus dT-QX significantly inhibited the tumor growth as compared to those of shRNA alone after two rounds of treatment. Consistently, three out of four tumors in the combined treatment have a much smaller cluster size than those with shRNA alone (Figure 7b). On the other hand, intravenous injection of dT-QX alone without shRNA suppression showed no significant inhibition of the tumor growth as compare with that of PBS (Figure 7). These in vivo results demonstrated that TYMP suppression plus dT-QX treatment was able to control the aggressive progression of Bel-7402 tumors and suggested that a combined treatment had a therapeutic potential on tumors with high levels of TYMP and TK1.

## Discussion

Our results indicated that high levels of TK1 were responsible for the cytotoxicity of dT-QX and high levels of TYMP counteracted this activity. In Hep3B cells, the transient suppression of TK1 led to a significant reduction of dT-QX cytotoxicity (Figure 3) while the overexpression of TK1 in HL-7702 resulted in a pronounced cytotoxicity (Figure 4). Similarly, the overexpression of TK1 in Bel-7402 cells led to increased cytotoxicity of dT-QX (Figure 3). These results in combined with the ER accumulation of dT-QX implied that cytosolic TK1 played a significant role on the cytotoxicity of dT-QX in cells. In contrast, TYMP counteracted the activity of dT-QX, which was supported by the enhanced cytotoxicity of dT-QX observed with siRNA or shRNA suppression in Bel-7402 cells (Figures 3 and 6). The counteraction of TYMP was further supported by the difference in cytotoxicity observed between Hep3B and other liver cancer cells that had high levels of TYMP (Figure 1). The counteraction by TYMP on dT-QX may be attributed to the possible catabolism of the thymidine quinoxaline conjugate to inactive metabolites, of which a recombinant enzymatic study could provide further insights. Unfortunately, the exact molecular targets by dT-QX have not so far been identified. In addition, the conversion of dT-QX to activated metabolites by TK1 has not been observed as expected by HPLC analysis in the cell treatment lysates. These results suggested that there were additional unknown pathways and mechanisms besides TK1 and TYMP for the action of dT-QX in cancer cells, which are currently under investigations.

Different levels of dT-QX cytotoxicity among these liver cancer cell lines reflected a common challenge in cancer chemotherapy due to the heterogeneity of tumor cells (Figure 1). All cancer cell lines had consistently high TK1 expression as compared to the normal liver derived HL-7702 cell line (Figure 1c), which was validated by IHC on human normal liver versus tumor samples (Figure 5a), supported TK1 as a tumor-specific target. In contrast, levels of TYMP protein varied dramatically among liver cancer cells but remained at a low level in normal liver cells. Clinically, induced high levels of TYMP have been commonly observed in tumor tissues due to inflammatory infiltration or after radiotherapeutic treatment and chemotherapy such as paclitaxel, doxorubicin and oxaliplatin [18,35,36]. Thus, high levels of TYMP in liver tumors are important subtypes and/or variations of liver cancers that need to be addressed specifically due to TYMP as a growth factor in tumors [14-16]. Our results showed that induced overexpression of TK1 via viral particles was unfortunately an ineffective approach to enhance the selective activity of thymidine analog due to induced cytotoxicity in the normal cells (Figure 4c vs 4d). Overexpression of herpes TK1 via viral gene delivery has been shown to increase the efficacy of nucleoside analogs in HCC models [38,39], while our data suggested that nonspecific cytotoxicity might concur in the liver cells. More importantly, our in vitro studies demonstrated that the suppression of TYMP by shRNA significantly enhanced the selectivity of thymidine analog dT-QX on cancer cells that have high levels of TYMP and TK1 (Figure 6). In addition, our in vivo subcutaneous Bel-7402 tumor model further supported the effectiveness of this approach (Figure 7).

The potential of this combination strategy has recently manifested by the result from clinical phase II trial of TAS-102 on colorectal cancer [40], although additional studies on refractory subtype are needed [41]. TAS-102 is a combination of antimetabolite *α,α,α*-trifluorothymidine plus a potent TYMP chemical inhibitor. Trifluorothymidine is activated via cytosolic TK1 phosphorylation to block thymidylate synthase [42] yet is highly toxic and has short plasma half-life [18]. The efficacy of TS-102 has been shown to correlate with the ratio of TK1/TYMP [42] and had limited responses in patients with solid tumors [43,44]. In contrast, our dT-QX selectively blocked cellular DNA synthesis in liver cancer cells with subsequent mitochondrial superoxide stress, possibly via DNA intercalation [29]. More importantly, our results in this study indicated that TYMP alone was a critical target to enhance the selectivity of a thymidine conjugate on cancer cells.

## Conclusions

Our study demonstrated that TK1 was responsible for anticancer activity of thymidine conjugates while TYMP as the thymidine metabolic enzyme was responsible for the varied biological activity. By taking advantage of low levels of TK1 and TYMP in normal liver tissue, the use of anticancer thymidine conjugate combined with TYMP suppression could directly target thymidine salvage pathway in liver cancer cells with various levels of TYMP addressed as tumor heterogeneity to be fully inhibited. Thus, the treatment of thymidine conjugate combined with TYMP suppression could be a promising direction to control the aggressive growth of liver tumors that had high levels of TYMP and TK1. This strategy may well be expanded in the applications of other thymidine analogs used for cancer diagnosis and therapeutics.

## Availability of supporting data

The data supporting the results of this article are included within the article and its additional files.

## Additional files

Additional file 1: Figure S1. Fluorescence spectra of dT-QX and images in cellular accumulation studies. (a) The excitation and emission spectra were obtained with a 0.5 mM dT-QX methanol solution with Hitachi F-4500 fluorescence spectrometer (Tokyo, Japan) at room temperature; the maximum fluorescence excitation and emission wavelengths are at 398 and 483 nm, respectively. (b) Fluorescence images of intracellular accumulation of dT-QX in HepG2 and HL-7702 cells with co-staining of ER Tractor Red dye. Cells were treated with either DMSO (top panels) or 50 μM dT-QX (bottom panels) for 5 h and then stained with ER tracker Red. (c) Fluorescence images of intracellular accumulation of dT-QX in Hep3B, HepG2 and HL-7702 cells with ER-specific GFP expression. Cells were first treated with Beckmam ER-GFF transfect agent for 24 h and then treated with either DMSO (top panels) or 50 μM dT-QX (bottom panels) for 5 h and then images were captured with fluorescence microscope.
Additional file 2: Figure S2. Modulation of TYMP and TK1 by siRNA suppression in HepG2 cells. Western blot analysis of TYMP and TK1 expression in HepG2 cells was carried out at 48 h post siRNA suppression. Cell viability was obtained after 24 h treatment with dT-QX at 48 h post siRNA suppression (*P < 0.05 as compared to those under the same dT-QX concentration in cells alone).
Additional file 3: Figure S3. Western blot analysis of TYMP/TK1 expression in mouse tumor tissues at 72 h post intratumoral injection of control or TYMP shRNA plasmid complex.

## Abbreviations

TK1: Thymidine kinase 1
TYMP: Thymidine phosphorylase
HCC: Hepatocellular carcinoma
dT-QX: Thymidine quinoxaline conjugate
ER: Endoplasmic reticulum
IHC: Immunohistochemical
