# Supplementary figures and images for: Anticancer activity of a thymidine quinoxaline conjugate is modulated by cytosolic thymidine pathways
Source: BMC Cancer. 2015 Mar 21;15:159. doi: 10.1186/s12885-015-1149-5 (PMC4374574; doi:10.1186/s12885-015-1149-5)

a)

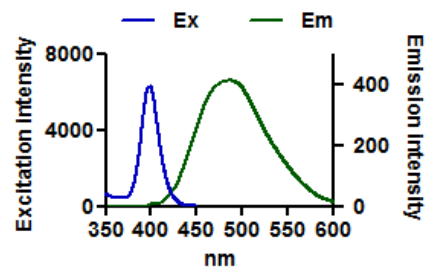

b)

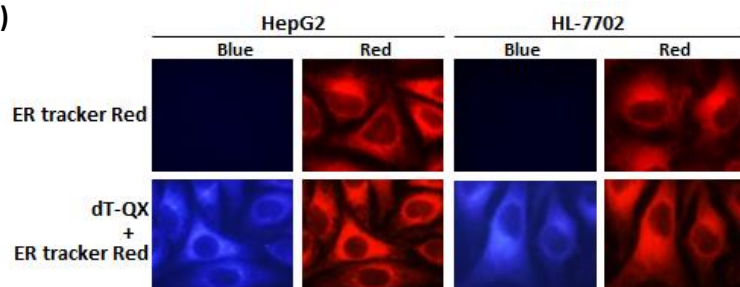

c)

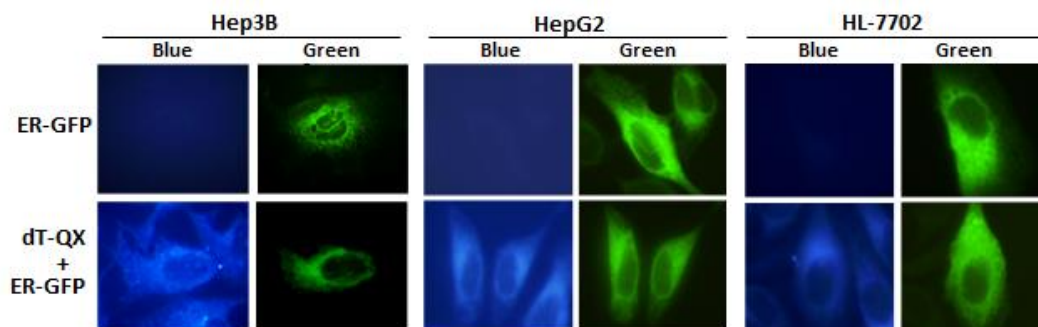

Supplement: Additional file 1: Figure S1. — Fluorescence spectra of dT-QX and images in cellular accumulation studies. (a) The excitation and emission spectra were obtained with a 0.5 mM dT-QX methanol solution with Hitachi F-4500 fluorescence spectrometer (Tokyo, Japan) at room temperature; the maximum fluorescence excitation and emission wavelengths are at 398 and 483 nm, respectively. (b) Fluorescence images of intracellular accumulation of dT-QX in HepG2 and HL-7702 cells with co-staining of ER Tractor Red dye. Cells were treated with either DMSO (top panels) or 50 μM dT-QX (bottom panels) for 5 h and then stained with ER tracker Red. (c) Fluorescence images of intracellular accumulation of dT-QX in Hep3B, HepG2 and HL-7702 cells with ER-specific GFP expression. Cells were first treated with Beckmam ER-GFF transfect agent for 24 h and then treated with either DMSO (top panels) or 50 μM dT-QX (bottom panels) for 5 h and then images were captured with fluorescence microscope. [file 12885_2015_1149_MOESM1_ESM.pdf]

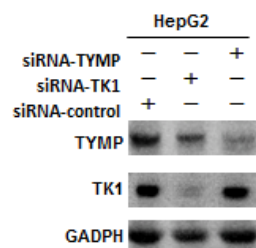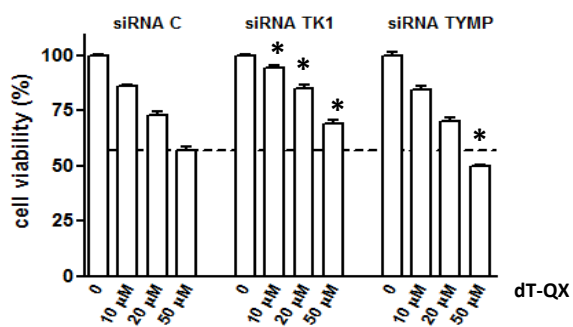

Supplement: Additional file 2: Figure S2. — Modulation of TYMP and TK1 by siRNA suppression in HepG2 cells. Western blot analysis of TYMP and TK1 expression in HepG2 cells was carried out at 48 h post siRNA suppression. Cell viability was obtained after 24 h treatment with dT-QX at 48 h post siRNA suppression (*P < 0.05 as compared to those under the same dT-QX concentration in cells alone). [file 12885_2015_1149_MOESM2_ESM.pdf]

tumor tissue

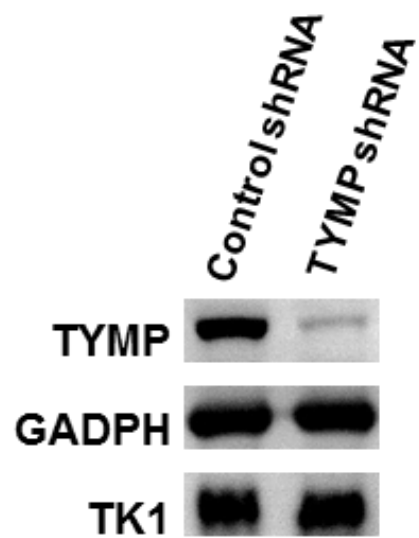

Supplement: Additional file 3: Figure S3. — Western blot analysis of TYMP/TK1 expression in mouse tumor tissues at 72 h post intratumoral injection of control or TYMP shRNA plasmid complex. [file 12885_2015_1149_MOESM3_ESM.pdf]
